# *Camellia puhoatensis* (Sect. Archecamellia – Theaceae), a new species from Vietnam

**DOI:** 10.3897/phytokeys.153.49388

**Published:** 2020-07-16

**Authors:** Danh-Hùng Nguyễn1, Văn-Dũng Lương2, Thi-Hương Lê3, Quốc-Thành Trần4, Ngọc-Đài Đỗ1,5, Ngọc-Sâm Lý1,6

**Affiliations:** 1 Graduate University of Science and Technology, VAST, 18 Hoang Quoc Viet, Cau Giay District, Ha Noi, Vietnam Graduate University of Science and Technology Ha Noi Vietnam; 2 Faculty of Biology, Da Lat University, 1 Phu Dong Thien Vuong Road, District 8, Da Lat City, Lam Dong Province, Vietnam Da Lat University Da Lat Vietnam; 3 School of Natural Science Education, Vinh University, 182 Le Duan, Vinh City, Nghe An Province, Vietnam Vinh University Vinh Vietnam; 4 Department of Science and Technology Nghe An, 75, Nguyen Thi Minh Khai, Vinh City, Nghe An Province, Vietnam Department of Science and Technology Nghe An Vinh Vietnam; 5 Faculty of Agriculture, Forestry and Fishery, Nghe An College of Economics, 51 Ly Tu Trung, Vinh City, Nghe An Province, Vietnam Nghe An College of Economics Vinh Vietnam; 6 Department of Biological Resources, Institute of Tropical Biology, VAST, 85 Tran Quoc Toan, District 3, Ho Chi Minh City,Vietnam Institute of Tropical Biology Ho Chi Minh Vietnam

**Keywords:** *
Camellia
*, section *Archecamellia*, taxonomy, Theaceae, Vietnam

## Abstract

A new species of Theaceae, *Camellia puhoatensis* N.S. Lý, V.D. Luong, T.H. Le, D.H. Nguyen & N.D. Do, **sp. nov.**, is described and illustrated from Nghe An Province, North Central Coastal Region, Vietnam. It is most similar to *C. chrysanthoides*, *C. flavida* and *C. petelotii* within sect. Archecamellia in shape and colouration of leaf, petal, ovary and glabrous stamen, but differs by its young puberulous shoot, mature leaf sparsely puberulous abaxially and leaf base rounded or broadly obtuse, petiole and pedicel puberulous, tepals 12–13, ovary and style pubescent. The comparison between the new species and *C. velutina* and *C. dormoyana* is presented. Data on distribution, ecology, phenology, use and provisional conservation assessment of the new species are given along with an illustration and a colour plate.

## Introduction

*Camellia*Linnaeus (1753) is the largest genus of the family Theaceae, with recent authors recognising species between 120 (Ming and Bartholomew 2007) and 280 (Chang 1981; Gao et al. 2005), distributed widely in East and Southeast Asia, from the Himalayas east to Japan and Indonesia (Chang and Ren 1998; Ming and Bartholomew 2007). The highest species diversity is found in China and Vietnam (Chang and Ren 1998; Ming 2000; Orel and Wilson 2012b). *Camellia* is distinguished from other genera of Theaceae by its usually large and apically dehiscent capsules and wingless (semi-)globose or polygonal seeds with an umbilicate hilum (Ming and Bartholomew 2007). The general introduction to the genus, with particular focus on Vietnam, was given in recent publications by various authors (e.g. Orel et al. 2012, 2013, 2014a, b; Luong et al. 2016a; Nguyen et al. 2018; Pham et al. 2019; Do et al. 2019a, b). So far, more than 75 species of *Camellia* have been reported in Vietnam, with many localised endemic species (e.g. Pitard 1910; Gagnepain 1941; Rosmann 1999; Tran 1998a, b; Pham 2000; Hakoda and Tran 2001; Hakoda et al. 2007; Orel 2006; Orel and Wilson 2010a, b, 2012a, b; Orel and Curry 2014; Orel et al. 2012, 2013, 2014a, b; Tran et al. 2012; Tran and Luong 2012, 2013; Tran and Le 2013, 2015; Luu et al. 2015, 2018; Luong et al. 2016a, b; Le et al. 2017; Nguyen et al. 2018; Pham et al. 2019; Do et al. 2019a, b), but the actual number is expected to be higher in the near future (Le and Luong 2016, Do et al. 2019b).

During recent extensive floristic surveys in the North Central coastal region in Vietnam, several interesting species of *Camellia* in yellow flower were collected by one of us (N.-D. Do) and colleagues in 2018–2019 (e.g. Tran and Luong 2013; Tran and Le 2015; Luong et al. 2016a, b; Le et al. 2017; Nguyen et al. 2018; Pham et al. 2019; Do et al. 2019a, b). Critical examination of living flowers, dried specimens and comparison with type material and protologues of all related yellow *Camellia* in Vietnam and China (e.g. Sealy 1958; Chang 1981; Chang and Bartholomew 1984; Gao et al. 2005; Ming 2000; Ming and Bartholomew 2007; Pham 2000; Orel and Curry 2014) led to the discovery of several new taxa, two of which were recently described and named *C. pukhangensis* D.N. Do, D.V. Luong, S. T. Hoang & H.T. Le and *C. ngheanensis* N.D. Do, V.D. Luong, N.S. Ly, T.H. Le & D.H. Nguyen (Do et al. 2019a, b), while some other collections are still awaiting description. In this paper, we describe a further new *Camellia* from the Pu Hoat Nature Reserve, Nghe An Province, Vietnam. The overall plant habit, somewhat ovate leaf blades, orbicular sepals and bright yellow tepal of these plants in Pu Hoat NR show similarities with *C. chrysanthoides* H.T. Chang, *C. flavida* H.T.Chang, *C. petelotii* (Merr.) Sealy and *C. dormoyana* (Pierre) Sealy (Sealy 1949, 1958; Chang 1979, 1981). However, it shows significant differences in its vegetative and floral structures (see Table 1) and we describe it here as a new species to science, *C. puhoatensis*.

## Materials and methods

The descriptions are mainly based on measurements from mature individuals of living plants in the field, supplemented by measurements from herbarium specimens. Type specimens of the most closely-related species of yellow camellias were examined from the following herbaria: DLU, HN, P, NSW and VNM (herbarium codes follow Thiers 2018). Hi-resolution digital images available were also accessed from botanical websites (e.g. https://science.mnhn.fr/, http://www.cvh.org.cn/, https://avh.ala.org.au/, https://plants.jstor.org/). All morphological characters were described using the general terminology and standard works of Sealy (1958), Chang (1981), Chang and Bartholomew (1984), Chang and Ren (1998), Gao et al. (2005), Ming (2000) and Ming and Bartholomew (2007). The conservation status was assessed, based on field observations in accordance with the IUCN Red List Categories and Criteria version 3.1 (IUCN 2017).

## Taxonomic treatment

### Camellia puhoatensis

Taxon classificationPlantaeEricalesTheaceae

N.S. Lý, V.D. Luong, T.H. Le, D.H. Nguyen & N.D. Do sp. nov.

44ED8C82-E5AF-5682-9C35-7A3549E0CA24

urn:lsid:ipni.org:names:77210595-1

Figures 1 , 2

#### Diagnosis.

*Camellia puhoatensis* is morphologically similar to *C. chrysanthoides*, *C. flavida* and *C. petelotii*, but differs in having young puberulous shoots, mature leaves sparsely puberulous abaxially with leaf bases rounded or broadly obtuse, petioles and pedicels puberulous, tepals 12–13 and the ovary and styles pubescent.

Type. VIETNAM. Nghe An Province: Que Phong District, Dong Van Commune, Pu Hoat NR, 19°43'31"N, 105°05'43"E, 270 m elev., 30 December 2018, *Do Ngoc Dai, Le Thi Huong, Nguyen Danh Hung, DHH-682* (holotype VNM; isotypes P, DLU).

**Figure 1.**
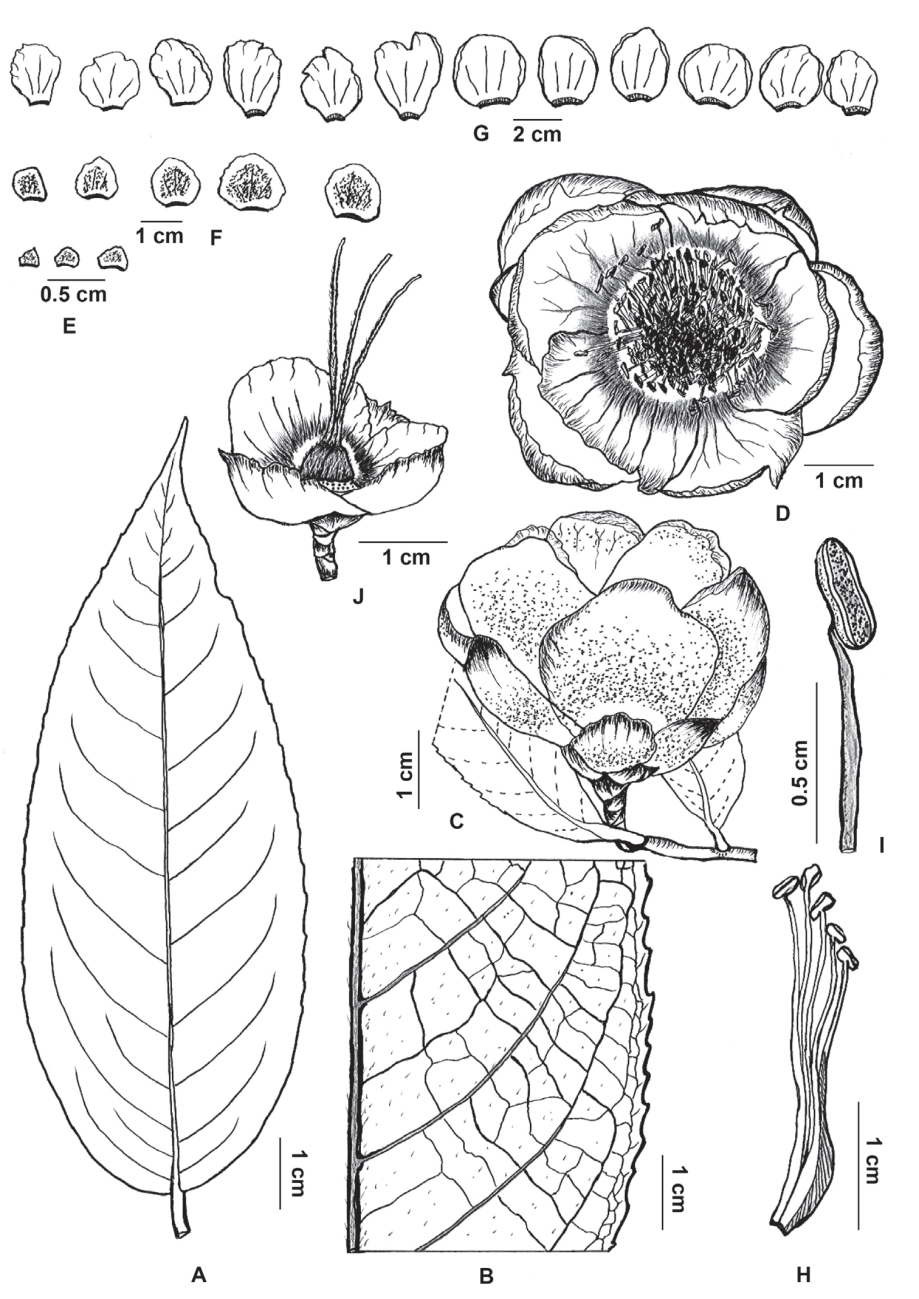
*Camellia puhoatensis*. **A** Leaf, adaxial view **B** venation detail of leaf (abaxial surface) **C** flower (lateral view) **D** flower (top view) **E** bracteoles (inner surfaces shown) **F** sepals (adaxial surfaces) **G** petals (adaxial surfaces) **H** androecium (one part) **I** stamen **J** gynoecium (with sepals and petals). Drawn from the holotype by Van-Dung Luong.

#### Description.

Shrub to tree, 2–7 m tall; *young shoots* puberulous then glabrous when aging, purple towards terminals; semi-mature branches brown, smooth, glabrous, leaf scars prominent; *adult branches* and trunk light grey, smooth with lighter-coloured patches and covered by lichens; *axillary leaf buds* rudimentary, roughly triangular, flat, with rounded apex, pubescent, brown, bud scales small but prominent, 1–3 mm long. *Leaves*: juvenile leaves forming a narrow tube when young, soft, somewhat pendulous, purple in colour; young leaves slightly serrate, shiny, purple, adaxially glabrous, abaxially puberulous; developing leaves descending, narrow, shiny, purple to green-purple tinted, abaxial surface puberulous; mature leaves serrate, irregularly towards the apex, 17–23 × 5.0–6.5 cm; petiole 8–16 × 4–5 mm, puberulous; lamina thin, coriaceous, oblong ovate or oblong, leaf apex acuminate or narrowly acuminate, base rounded or broadly obtuse, adaxially dark green and glabrous, abaxially pale green and sparsely puberulous; primary vein continues as a shallow channel on the adaxial side of the petiole, 2.0–2.5 mm wide proximally, less than 1.0 mm distally, proximally light green and shiny on both sides; secondary venation pinnate, indistinctly brochidodromous, partially eucamptodromous on some leaves, with 10–13 pairs; midribs and lateral veins sunken adaxially; veins distinct proximally, less so towards the apex and the margins; tertiary venation very indistinct, sometimes lacking, more prominent at the leaf margins. *Flowers* usually solitary, sometimes together in groups of 2 flowers borne on a short bracteate shoot, terminal, rarely axillary, lacking scent, 4.5–6.0 cm in diameter; *pedicel* stout, covered by purplish-red perulae, 7–10 mm long, puberulous; flower buds unevenly globose in shape, 2.2–2.6 × 2.0–2.3 cm, yellowish-red tinted, open flowers somewhat circular; *bracteoles* (sensu Sealy 1958) 3–4, opposite, orbicular, 1.5–2.5 × 1.5–3.0 mm, abaxially red to yellow-red tinted, adaxially paler, glabrous, margins ciliate, persistent; *sepals* 5, persistent, orbicular or subglobose, 0.6–1.5 × 0.8–1.8 cm, abaxially dull red and pubescent, adaxially pale yellowish and glabrous, margins ciliate; *petals* 12–13, arranged in 3 whorls, bright yellow, sometimes with large red patch on the outer ones; outermost whorl comprising 3 or 4 petals, orbicular to broadly obovate, 2.2–2.8 × 1.6–2.3 cm, abaxially pubescent, adaxially glabrous; middle whorl comprising 4 or 5 petals, broadly obovate, 2.4–3.3 × 1.8–2.5 cm, abaxially pubescent, adaxially glabrous; innermost whorl of 3 or 4 petals, orbicular to broadly obovate, 2.3–2.5 × 1.7–2.2 cm, abaxially pubescent, adaxially glabrous, basally united with outermost filaments 5–7 mm. *Androecium* numerous stamens, in 4–5 whorls, light yellow, 2.5–2.8 cm long, glabrous; outer filaments basally united for 1.5–1.8 cm forming a cup, inner ones basally united for 3–5 mm, free above union; *anthers* yellow, 2.2–2.8 × 1–1.5 mm, with two longitudinal striations, dorsifixed. *Gynoecium* superior, 3–(4)-loculed, ovoid terminating into 3–(4) styles, 2.5–3.0 × 3.0–3.5 mm, slightly longitudinal striations, pubescent, 2 ovules per locule; *styles* free to the base, 1.8–2.3 cm long, pubescent. *Capsule* not seen.

**Figure 2.**
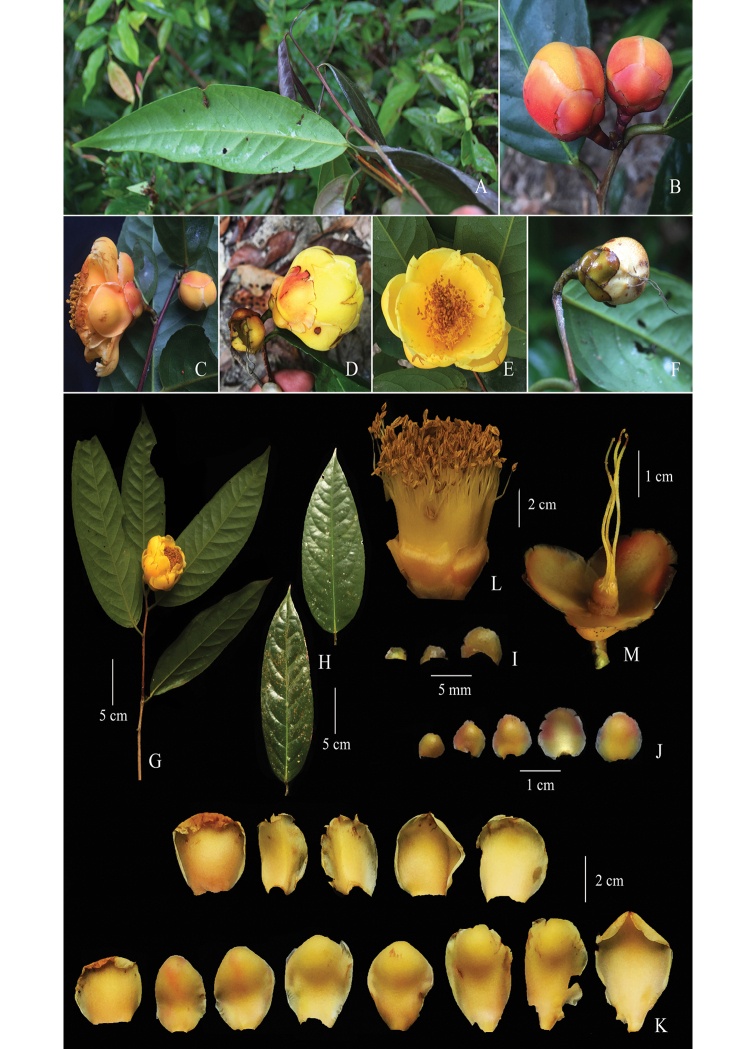
*Camellia puhoatensis*. **A** young shoot **B** terminal buds **C** solitary bud and axillary flower (side view) **D** flower and pollinated flower (side view) **E** close-up of flower (front view) **F** immature fruit **G** a part of branch showing leaves abaxial and opening flower **H** leaves adaxially **I** bracteoles **J** sepals **K** petals **L** androecium with stamens **M** gynoecium (with sepals and styles). Photos by Ngoc-Dai Do, the colour plate prepared by Ngoc-Sam Ly.

#### Phenology.

Flowering from November to January of the next year.

#### Distribution and habitat.

*Camellia puhoatensis* is currently found only from the type locality. It grows on moist fertile and sandy soils along mountain streams and hill slopes in evergreen broad-leaved forests in Pu Hoat Nature Reserve, Vietnam, at elevations of 270–450 m.

#### Provisional conservation assessment.

At present, only a single population of about 300 scattered mature individuals of *Camellia puhoatensis* was observed in the type locality, with an estimated extent of occurrence (EOO) less than 100 km^2^ and an area of occupancy (AOO) less than 1 km^2^. The population is highly threatened due to loss of habitat within its range and high market demands for wild, yellow-flowered camellias which are intensively collected for sale by local people. Therefore, *C. puhoatensis* is preliminarily categorised as Critically Endangered [B1ab (i, ii, iii) + 2ab (ii, iii), D], according to the IUCN Categories and Criteria (IUCN 2017).

#### Etymology.

The specify epithet ‘*puhoatensis*’ refers to the type locality.

#### Uses.

Leaves and flowers were harvested and used for tea by the local people.

#### Additional specimens examined.

***Paratypes.*** Vietnam. Nghe An Province: Que Phong District, Dong Van Commune, Pù Hoạt NR, 19°48'45"N, 105°5'39"E, 320 m elev., 2 September 2018, *Đỗ Ngọc Đài, Nguyễn Danh Hùng, Lê Thị Hương*, *DHH 120* (VNM); the same locality, 19°48'31"N, 105°05'43"E, 280 m elev., 16 January 2019, *Đỗ Ngọc Đài, Nguyễn Danh Hùng, Lê Thị Hương*, *DHH 790* (DLU), *DHH* 791 (HN).

#### Vernacular name.

Vietnamese language: Trà hoa vàng pù hoạt.

#### Taxonomic notes.

The current infrageneric classification of *Camellia* is derived from three previous publications (Sealy 1958; Chang and Bartholomew 1984; Ming 2000) and was based on the assessment of morphological characteristics. The taxonomic system of Sealy (1958) and Chang and Bartholomew (1984) are used to describe and determine the placement of new taxa within *Camellia*. These systems are the most detailed and comprehensive study of the genus and also provide the basis for our current understanding of the genus. The taxonomic system of Ming (sensu Ming and Bartholomew 2007) was used for supplementary data only as it appears to be superficially similar to the system of Sealy (Orel and Wilson 2010a). In this paper, we have followed the Sealy’s (1958) taxonomic system to consider the placement of the new species within Section Archecamellia Sealy of *Camellia*. Morphologically, *C. puhoatensis* possesses a solitary or paired flowers at terminal (sometimes axillary), stout, thick and erected pedicel, 3–4 persistent bracteoles, 5 persistent sepals (undifferentiated bracteoles and sepals), large yellow flowers with 12 tepals that are inner ones basally connate and adnate to androecium, androecium free above the union with the petals or unified for some distance to form a fleshy cup, filaments glabrous, gynoecium 3(–4)-loculi, styles 3(–4) and free to the base. These characteristics are not only identical to the species of sect.Archaecamellia Sealy (sensu Sealy 1958; sensu Chang and Bartholomew 1984), but also share with species of sect.Stereocarpus which possesses 2 or 4 bracteoles (sensu Sealy 1958), terminal flowers (sensu Chang and Bartholomew 1984), stamens in 3–4 whorls, ovary with 3–5 locules (sensu Ming 2000), as well as sharing with species of sect.Chrysantha Chang, such as distinct peduncle, small floral bracteolates, yellow flowers, three carpels of gynoecium and separate styles (sensu Chang 1979). As characterized by Sealy (1958), sect. Archaecamellia shares several traits with sect. Stereocarpus. These include a solitary and erected flower at the end of the branches, persistent bracteoles and sepals, stamens united with the petals and glabrous filaments. However, traits that are distinctive to sect. Archaecamellia include (6–)11–16 indistinct bracteoles and sepals, 8–14 petals, glabrous or pubescent gynoecium and 3 or 5 free styles (vs. 2 or 4 bracteoles and 5 or 6 sepals, ca. 12 petals, glabrous gynoecium and a single style in sect. Stereocarpus). The sect. Chrysantha also shares several traits with sect. Archaecamellia in having yellow and pedicellate flowers, persistent bracteoles and sepals, glabrous or hairy filaments and gynoecium, but it can be distinguished from sect. Archaecamellia by the axillary flowers, distinct bracts and sepals and 3–5 cleft styles (Chang 1979). Section Archaecamellia is currently comprised of 19 species (Sealy 1958; Ming 2000; Orel and Wilson 2012a; Do et al. 2019a). The new species is most similar to *C. chrysanthoides* H.T.Chang, *C. flavida* H.T.Chang and *C. petelotii* (Merr.) Sealy in having the same plant habit, somewhat oblong leaves, yellow flowers, glabrous 3-loculed gynoecium with 3 styles free to the base. A detailed morphological comparison between *C. puhoatensis* and these three species is provided in the above diagnosis and in Table 1. Moreover, *C. puhoatensis* also resembles *C. dormoyana* (Pierre) Sealy of sect. Stereocarpus (Sealy 1958) and *C. velutina* V.T. Pham et al. of sect. Chrysantha (Pham et al. 2019) by somewhat oblong leaves, yellow flowers and glabrous stamens. However, *Camellia dormoyana* is easily distinguished from *C. puhoatensis* by having the young shoots, mature leaves and petioles all glabrous, the sessile pedicel and 5–6 bracteoles abaxially velutinous, the abaxial petals silky velutinous, the ovary being glabrous and with five locules and the styles united for their entire length and glabrous. Similarly, *C. velutina* is readily distinguished from *C. puhoatensis* by its glabrous young shoots, mature leaves and petioles, sepals that are silky velutinous abaxially and velutinous adaxially, the 10 (occasionally 11) petals that are silky velutinous and glabrous ovary and style (see Table 1).

**Table 1.** Morphological comparison of *C. puhoatensis* with its most closely-related taxa (based on Sealy 1958; Chang and Bartholomew 1984; Tran and Hakoda 1998; Pham et al. 2019).

Characters	*C. puhoatensis*	*C. chrysanthoides*	*C. flavida*	*C. petelotii*	*C. velutina*	*C. dormoyana*
Young shoot	puberulous	glabrous	glabrous	glabrous	glabrous	glabrous
Leaf blade	oblong ovate or oblong, 17–23 × 5–6.5 cm, base rounded or broadly obtuse, abaxially sparsely puberulous	oblong, 10–18 × 3.0–6.5 cm, base cuneate, glabrous	elliptic to oblong, 6.0–10 × 2.1–4.5 cm, base broadly cuneate, glabrous	broadly oblong or oblong-oval, 14.5–18 × 4.5–7.5 cm, base broadly cuneate, glabrous	oblong to elliptic, 15–22 × 5–11 cm, base broadly cuneate to rounded, glabrous	oval or oblong or ovate, 11–18(–25) × 5.5–8.5 cm, base cuneate to rounded, glabrous
Petiole	puberulous	glabrous	glabrous	glabrous	glabrous	glabrous
Flower	solitary (2 flowers), terminal, rarely axillary	solitary, mostly axillary	solitary, terminal and axillary	solitary, terminal	solitary, terminal or axillary	solitary, terminal
Pedicel	7–10 mm long	3–4 mm long	1–2 mm long	10–12 mm long	10–13 mm	sessile
Bracteoles	3–4, glabrous	4–6, abaxially pubescent	4–5, glabrous	(6–)8–10, abaxially puberulous	2(–3), abaxially velutinous	5–6, abaxially silky velutinous
Sepals	5, abaxially pubescent	5, abaxially puberulent	4–6, glabrous	5, abaxially puberulous	5, adaxially velutinous	abaxially silky velutinous
Petals	12–13, abaxially pubescent	8–9, abaxially puberulent	8, glabrous	ca. 14, abaxially puberulous	10(–11), velutinous	12, silky velutinous
Stamen	glabrous	glabrous	glabrous	glabrous	glabrous	glabrous
Ovary	3–(4) loculi, ovoid, pubescent	ovoid, 3 loculi slightly tomentose	globose, 3 loculi, glabrous	globose, 3 loculi, glabrous	3 loculi, ovoid, glabrous	globose, 5 loculi, glabrous
Style	3–(4), free to base, pubescent	3, free to base, glabrous or sparsely pubescent	3, free to base, glabrous	3, free to base, glabrous	3, free to base, glabrous	5, united, glabrous

## Supplementary Material

XML Treatment for Camellia puhoatensis
